# Inhibitory effects of tibial nerve stimulation on bladder neurophysiology in rats

**DOI:** 10.1186/s40064-016-1687-6

**Published:** 2016-01-15

**Authors:** Mahipal Choudhary, Ron van Mastrigt, Els van Asselt

**Affiliations:** Department of Urology, Sector FURORE, Room EE1630, Erasmus MC, Dr. Molewaterplein 50, 3015 GE Rotterdam, The Netherlands

**Keywords:** Tibial nerve stimulation, Overactive bladder, Neuromodulation, Afferent nerve activity

## Abstract

Tibial nerve stimulation (TNS) is a form of peripheral neuromodulation which has been found effective in treating overactive bladder symptoms, with lesser side effects than first line pharmacotherapy. Despite its widespread clinical use, the underlying mechanism of action is not fully understood. Our aim was to study its effect on the bladder neurophysiology and the trigger mechanism of voiding in the overactive detrusor, simulated by acetic acid (AA) instillation. In urethane anaesthetized male Wistar rats, the tibial nerve was stimulated for 30 min at 5 Hz, pulse width 200 µs and amplitude approximately three times the threshold to induce a slight toe movement. The pressure at which a voiding contraction was triggered (p_thres_) did not change significantly between the pre- and post-TNS measurements in AA induced detrusor overactivity. It was found that TNS significantly reversed the effects of AA irritation by increasing the bladder compliance and the bladder volume at p_thres_, as well as suppressed the threshold afferent nerve activity. The slope of the linear relationship between pressure and the afferent activity increased after AA instillation and decreased significantly after stimulation. In addition to its well-known central inhibitory mechanisms, this study has demonstrated that TNS improves bladder storage capacity by delaying the onset of voiding, via an inhibitory effect on the bladder afferent signaling at the peripheral level.

## Background

A voiding reflex is initiated by bladder afferent nerve fibers which run through the major pelvic ganglion via the pelvic nerve to the L4–S3 level of the spinal cord. Bladder efferent fibers originate at the same level, travel via the spinal nerve, synapse in the major pelvic ganglion and innervate the bladder smooth muscle (Pascual et al. 1989; Keast et al. 1989). At the same spinal level the sciatic nerve originates which at the mid-thigh level splits into tibial, peroneal and sural nerves (Brunner et al. 1980). It is generally believed that superseding a threshold of afferent nerve activity triggers efferent firing to the bladder wall i.e. initiates a voiding contraction (van Asselt et al. 1999). In pathological conditions such as detrusor overactivity this afferent–efferent mechanism is disturbed (de Groat 1997). Evidence of this anomaly is the increased sensitivity of afferent fibers, particularly C-fibers, which has been reported to be one of the underlying causes of detrusor overactivity (Steers 2002). The increased sensitivity leads to symptoms like urgency and frequency. One treatment technique is based on stimulation of posterior tibial nerves for 30 min a few times a week for several months (Peters et al. 2013; Govier et al. 2001). Although this technique has been reported to be promising, the underlying mechanism of action is only partially understood (Gaziev et al. 2013).

To explain the inhibitory effect of tibial nerve stimulation on the bladder, most studies have focused on urodynamic parameters. As neuromodulation takes place via the afferent and/or efferent pathways, our aim was to study its effect on the bladder neurophysiology and the trigger mechanism of voiding in a detrusor overactivity rat model.

## Results

**Fig. 1:**
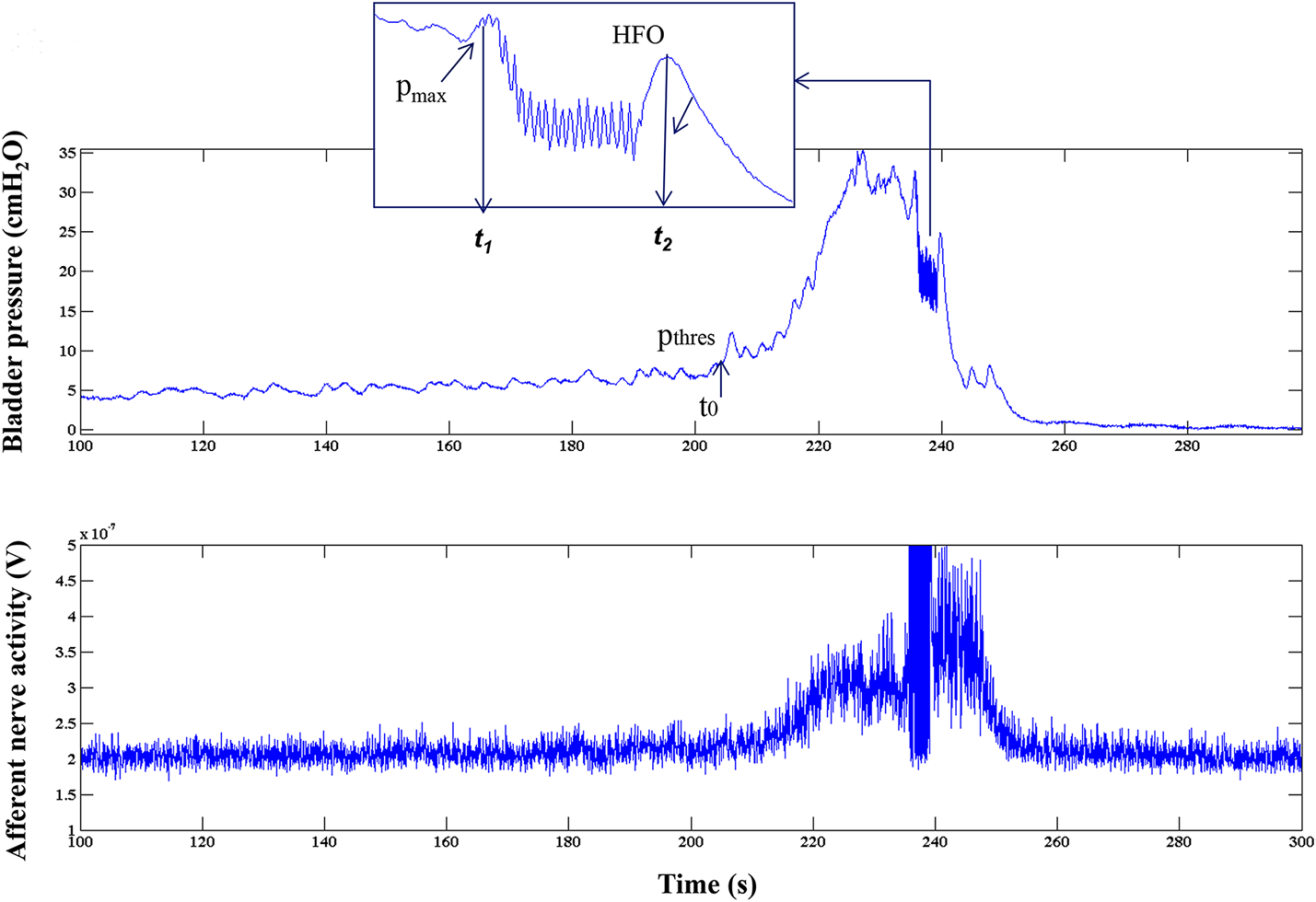
Nerve activity and bladder pressure development during a typical rat voiding cycle. This figure shows a part of a bladder filling cycle. The *upper panel* shows the pressure during the filling phase with voiding (t_1_ − t_2_), marked by high frequency oscillations (HFO) of the urethral sphincter. At p_thres_ the pressure rises above the baseline and increases rapidly until a voiding occurs. p_max_ represents the maximum pressure just before the start of urine flow. The *lower panel* shows the corresponding afferent nerve activity

**Fig. 2:**
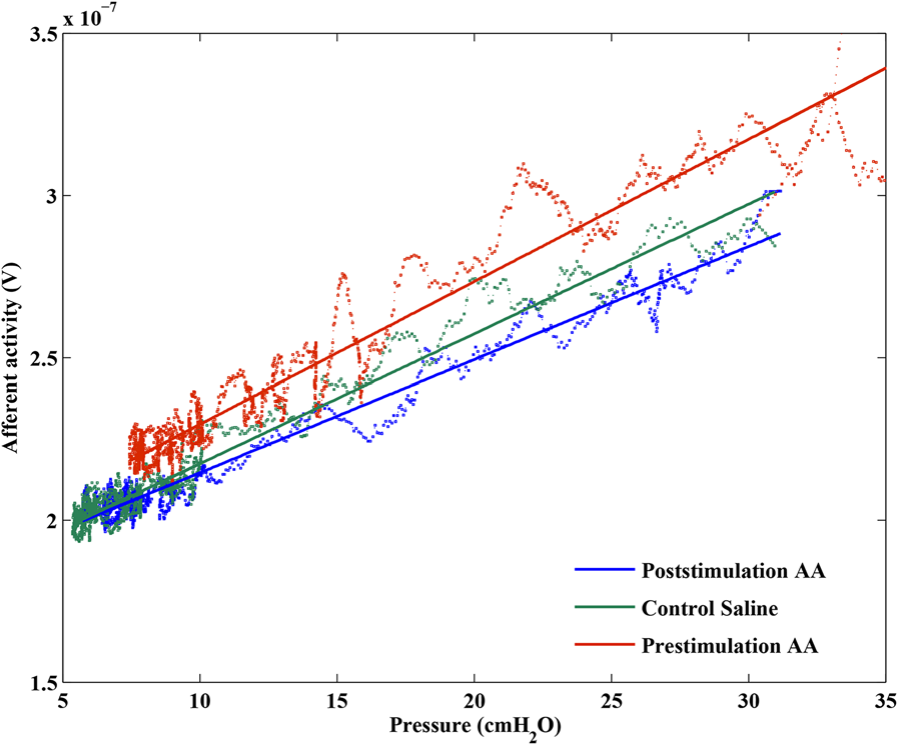
Example of the effect of tibial nerve stimulation on the slope of the pressure-afferent nerve activity. The slope of afferent nerve activity as a function of bladder pressure was fitted with a *straight line* representing a linear polynomial fit. Instillation of 0.5 % AA significantly increased the slope of this line in the prestimulation AA measurement (*topmost line*) as compared to the control saline measurement (*middle*) and decreased significantly after tibial nerve stimulation (*bottom*) (3 measurements within 1 animal)

No measurements could be done in 4 of the 14 rats due to equipment failure and the absence of bladder contractions. In the remaining 10 rats a few measurements were excluded from the analysis due to movement artifacts, or a high noise level caused by electrodes touching the surrounding tissue. These artefacts were manifested by irregular peaks in the pressure signal. The p_thres_ which marks the beginning of a voiding contraction did not vary among the saline, AA and poststimulation measurements (Table 1). Irritation of the bladder with AA induced an increase in the threshold afferent activity, which was significantly reversed in the poststimulation AA measurements. As expected, the threshold volume and bladder compliance were significantly reduced after AA irritation, and were also restored in the poststimulation AA measurements (Table 1). The maximum pressure (p_max_) just before the HFOs was found comparable between the three groups, whereas the corresponding afferent nerve activity increased on AA instillation and decreased poststimulation. The pressure rise time (t_0_ − t_1_, Fig. 1) from the p_thres_ to the actual voiding seemed higher in saline measurements, however the difference between the three groups was not statistically significant. The slope of the pressure–afferent nerve activity increased on AA instillation and decreased strongly after tibial nerve stimulation. An example of afferent nerve activity as a function of bladder pressure represented by a linear polynomial fit is shown in Fig. 2. The offset which represents nerve activity at pressure ~0 did not differ between the groups (Table 1). To validate the automatic calculation of pressure threshold and afferent activity by the custom written program, we also calculated these parameters by manual identification of the pressure threshold and the corresponding afferent threshold (Table 1). No statistical difference in results between the two methods was observed.

**Table 1:** Statistical comparison of estimated parameters in pre- and post-stimulation measurements

	Prestimulation saline (I)	Prestimulation AA (II)	Poststimulation AA (III)
Automatically calculated			
Threshold afferent activity (µV)	0.21 ± 0.02	0.23 ± 0.04^ф^	0.19 ± 0.01*
Maximum afferent activity (µV)	0.25 ± 0.04	0.41 ± 0.07^ф^	0.22 ± 0.04*
Threshold pressure, p_thres_ (cmH_2_O)	5.2 ± 2.4	4.4 ± 2.7^N.S.^	5.4 ± 2.8^N.S.^
Maximum pressure, p_max_ (cmH_2_O)	34.4 ± 7.0	32.5 ± 7.8^N.S.^	30.5 ± 5.2^N.S.^
Pressure rise time (s)	59.2 ± 55.9	54.6 ± 55.1^N.S.^	54.6 ± 47.9^N.S.^
Slope (µV/cmH_2_O)	1.6 ± 1.4	1.9 ± 1.5^ф^	0.7 ± 0.6*
Offset (µV)	0.21 ± 0.03	0.22 ± 0.04^N.S.^	0.19 ± 0.02^N.S.^
Volume, V_thres_ (mL)	0.84 ± 0.2	0.53 ± 0.2^ф^	0.76 ± 0.3*
Compliance (mL/cmH_2_O)	0.17 ± 0.03	0.10 ± 0.07^ф^	0.17 ± 0.06*
Manually calculated			
Threshold afferent activity (µV)	0.21 ± 0.02	0.23 ± 0.04^ф^	0.20 ± 0.21*
Threshold pressure (cmH_2_O)	6.5 ± 2.9	5.3 ± 2.3^N.S.^	6.7 ± 2.5^N.S.^
Rise time (s)	53.2 ± 55.9	48.6 ± 55.1^N.S.^	45.5 ± 33.9^N.S.^

Mean ± SD of the estimated parameters in control saline, prestimulation and poststimulation AA measurements, n = 72 measurements (saline = 18, prestimulation AA = 27, and poststimulation AA = 27). The symbols ф and * represent the statistical significance (p < 0.05) between group I vs II and II vs III respectively. N.S. indicates no statistical difference (p > 0.05) between the groups

## Discussion

Tibial nerve stimulation is a clinical alternative to pharmacotherapy and has been found effective in restoration of bladder capacity by inhibiting undesired detrusor contractions (Peters et al. 2013; Govier et al. 2001; van Balken et al. 2001). In addition, it has been shown to suppress simulated bladder overactivity in anaesthetized animal models (Tai et al. 2011; Su et al. 2015). To further the understanding of the working mechanism of TNS, we studied its effect on the pressure–afferent nerve activity relationship and the trigger mechanism of voiding.

A voiding reflex is initiated by bladder afferent firing superseding a certain threshold necessary to trigger efferent firing of the bladder nerves (van Asselt et al. 1999). This threshold has been defined as the point where pressure rises above the baseline and increases rapidly until voiding occurs. Although a change was expected during an inhibitory response, in our study, the pressure threshold did not change significantly between the pre- and post-tibial nerve stimulation measurements. However, the volume threshold was different between both groups after stimulation, implying that the stimulation delayed the onset of voiding to a higher filled volume, without affecting the pressure at this point.

The threshold afferent nerve activity and the slope of the linear pressure–afferent activity relation increased when the bladder was irritated by AA and decreased significantly poststimulation (Fig. 2). The increase in afferent activity can be ascribed to chemical irritation of bladder (Zvara et al. 2010), which causes hypersensitization of nociceptive C-fiber afferents (Tai et al. 2011). Whether the poststimulation decrease is a reversal of the C-fiber sensitization, cannot be ascertained due to the mixed, multifiber nature of our afferent recordings. Though urethane is believed to spare the micturition reflex (Matsuura and Downie 2000), a suppressing effect of anaesthesia on pressure development and/or afferent signaling cannot be ruled out, which however may be expected to affect the pre- and post-stimulation measurements to the same degree.

A peripheral effect of TNS on afferent nerve terminals, to the best of the authors’ knowledge, has not been reported elsewhere. It is possible that this effect is caused by a change in bladder compliance. Poststimulation the volume threshold increased while the pressure threshold remained unchanged, implicating an increase in detrusor compliance. It has been shown that pudendal nerve stimulation, via sympathetic efferent pathways, can activate β3-adrenoceptors in the detrusor muscle and/or α-adrenergic receptors at the vesical ganglia to inhibit detrusor contractions and regulate bladder compliance (McGee et al. 2014; Craggs and McFarlane 1999; Lindstrom et al. 1983; De Groat and Saum 1972). Similar hypotheses have been proposed for the working mechanism of tibial stimulation. According to Matsuta et al. (2014) “TNS might activate a reflex output to the bladder through the sympathetic hypogastric nerves and relax the detrusor via β-adrenergic mechanisms”. Further studies on the role of different nerve types in TNS inhibition are warranted for a better understanding.

The role of the central nervous system in neuromodulation has been well established in various studies (Zhang et al. 2015; Xiao et al. 2014; McGee et al. 2014; van der Pal et al. 2006). As the posterior tibial nerve projects to the sacral micturition center and the nucleus of Onuf, the same area where bladder projections are found, it is generally accepted that TNS evokes a central inhibition of micturition pathways and the therapeutic effect of TNS takes place via these areas (Bemelmans et al. 1999; Vandoninck et al. 2003). Recent studies have also suggested the involvement of inhibitory neurotransmitter mechanisms for TNS inhibition of bladder overactivity modulated by activation of μ, κ and δ opioid receptors (Zhang et al. 2015). As evident from the multitude of suggested modes of action, the working mechanism of tibial nerve stimulation is highly complex and occurs at several levels, incorporating a combination of central inhibitory mechanisms as well as an inhibition at the peripheral level, as suggested by our present study.

Although the results presented in this study underline some important basic neurophysiology behind tibial nerve stimulation, the large differences between rats and humans require a careful consideration of the translational aspects. Sensory urgency in humans is a subjective urodynamic diagnosis and cannot be simulated in animal models; therefore the acetic acid irritation model used in this study only mimics one symptom of the human overactive bladder syndrome, the detrusor overactivity.

## Conclusions

We studied the effect of tibial nerve stimulation on the neurophysiology of voiding in terms of the linear relationship between bladder pressure and afferent nerve activity. It was found that 30 min of stimulation favorably affected the slope of this linear relationship, by inhibiting bladder afferent activity and increasing bladder compliance in an anaesthetized rat model of detrusor overactivity. In addition to the established central inhibitory mechanism of TNS, our study provides evidence for an inhibitory effect on afferent signaling at the peripheral level. Additional studies in unanaesthetized animals and the effect of TNS on different neural pathways are warranted for a comprehensive understanding of the neurophysiological mechanism behind TNS.

## Methods

### Ethics, consent and permissions

All laboratory and experimental procedures were conducted in accordance with institutional guidelines of the local Erasmus MC Animal Experiment Committee [Dier Experimenten commissie (DEC)], DEC number: EMC 2092 and 3164, Protocol number: 102-10-06.

### Experimental procedures

A total of 14 male Wistar rats (432 ± 45 g) were used in this study. The animals were anesthetized with urethane (1.2 g/kg body weight, intraperitoneally) and placed on a heated undercover. The abdomen was surgically opened and a branch of the postganglionic pelvic nerve (crushed between the major pelvic ganglion and the electrode, so that only afferent nerve signals were recorded) was mounted on a custom made bipolar electrode consisting of two thin platinum/iridium hook shaped wires to record afferent nerve traffic. Since we only crushed one branch of the postganglionic pelvic nerve, leaving intact all other branches, this did not have a significant effect on the bladder function (Choudhary et al. 2015). A catheter connected to a disposable pressure transducer and infusion pump was inserted through the apex of the bladder dome. It was used to fill the bladder with saline (0.06 ml/min) and to record pressure (Statham SP1400 pressure monitor). The tibial nerve was accessed via the medial side of the right hind leg near the ankle. For stimulation a bipolar cuff electrode was placed around the nerve. The abdominal cavity was filled with paraffin oil to prevent the tissue from drying out. At the end of the experiments, the animals were euthanized using intracardiac KCl.

Initially the bladder was filled with physiological saline (0.9 % NaCl) until voiding occurred and 3–4 micturition cycles were recorded. Next, the bladder was repeatedly filled with 0.5 % Acetic Acid (AA) to induce bladder overactivity. Thereafter the bladder was emptied and the tibial nerve was stimulated for 30 min with monophasic rectangular pulses of frequency 5 Hz, width 200 µs and an amplitude approximately 3 times the threshold to induce a slight toe movement. Then, AA infusion was repeated to study the poststimulation effect of TNS.

### Signal processing

Afferent nerve activity was amplified by a DISA 15C01 EMG amplifier (amplification range: 100–200,000) and band-pass filtered with a Krohn-Hite 3944 filter (Bessel, 4th order, 200–2000 Hz). Nerve activity and pressure signals were displayed in real-time on a computer screen using a custom written LabVIEW^®^ (National Instruments, USA) program and were sampled and stored at 25 kHz and 25 Hz respectively. A custom written MATLAB^®^ (Mathworks, USA) program was used to process and analyse the recorded signals. The nerve signal was rectified and averaged by taking the mean of 1000 samples, effectively reducing a 1 s interval to 25 data samples, similar to the pressure.

The pressure threshold at which a voiding contraction began was determined by another custom written MATLAB^®^ program. The program first smoothed the signal using a Savitzky–Golay filter and then calculated the second derivative of the pressure signal. The pressure at which the second derivative superseded a certain threshold was defined as the pressure threshold (p_thres_) and the afferent nerve activity at this threshold was calculated. Additionally, we also calculated the filled volume (V_thres_) and the bladder compliance, $\frac{{dV_{thres} }}{{dp_{thres} }}$ at the pressure threshold.

The automatically derived pressure threshold and afferent activity were compared to values derived manually at the point where the pressure started to increase rapidly (t_0_, Fig. 1). In rats, voiding is marked by rapid contractions of the urethral sphincter, seen as high frequency oscillations (HFO) in bladder pressure recording. In Fig. 1, t_1_ is the time at the start of HFO and p_max_ is the maximum pressure just before the start of HFO.

To compare the relationship between bladder pressure and afferent activity pre- and post-tibial nerve stimulation, a linear regression model was fitted to the afferent activity–pressure data in the interval t_0_-t_1_ to calculate slope and offset.

All data are presented as mean ± SD. To compare groups a two-way ANOVA followed by Bonferroni multiple comparison was done using the SPSS^®^ statistical package (version 21.0, SPSS Inc., Chicago, IL, USA). A p < 0.05 was considered significant.

## Abbreviations

TNS: tibial nerve stimulation
AA: acetic acid
HFO: high frequency oscillations
p_max_: maximum pressure
p_thres_: threshold pressure
V_thres_: threshold volume
